# Research priorities about stoma‐related quality of life from the perspective of people with a stoma: A pilot survey

**DOI:** 10.1111/hex.12585

**Published:** 2017-07-04

**Authors:** Gill Hubbard, Claire Taylor, Becca Beeken, Anna Campbell, Jackie Gracey, Chloe Grimmett, Abi Fisher, Gozde Ozakinci, Sarah Slater, Trish Gorely

**Affiliations:** ^1^ University of Stirling Stirling UK; ^2^ London North West Healthcare NHS Trust London UK; ^3^ University College London London UK; ^4^ Edinburgh Napier University Edinburgh UK; ^5^ Ulster University Coleraine UK; ^6^ University of Southampton Southampton UK; ^7^ University of St Andrews St Andrews UK; ^8^ NHS Glasgow and Clyde Glasgow UK

**Keywords:** colostomy, ostomy, public/patient involvement, research priorities, stoma, urostomy

## Abstract

**Background:**

There is a recognized need to include patients in setting research priorities. Research priorities identified by people with a stoma are rarely elicited.

**Objectives:**

To improve the quality of life of people with a stoma through use of evidence‐based practice based on research priorities set by patients.

**Design and Methods:**

Online pilot survey publicized in 2016 via United Kingdom stoma charities. People ranked nine stoma‐related quality of life topics in order of research priority.

**Participants:**

People 16 years of age and over who currently have or have had a stoma for treatment for any medical condition.

**Analysis:**

Distributions of the priority scores for each of the nine research topics were examined. Group differences were explored using either the Mann–Whitney U‐test or the Kruskal–Wallis test depending on the number of groups.

**Results:**

In total, 225 people completed the survey. The most important research priority was pouch leak problems and stoma bag/appliance problems followed by hernia risk. There were statistically significant differences in ranking research priorities between males and females, age, underlying disease that led to a stoma, stoma type and length of time with a stoma.

**Conclusion:**

People with a stoma are willing to engage in and set research priorities. The results should contribute towards future research about setting the research agenda for the study of stoma‐related concerns that impact quality of life.

**Key Points**

1. This pilot study addresses the lack of patient‐led research priority setting about stoma‐related quality of life.
2. The most important research priority was pouch leak problems and stoma bag/appliance problems. The second research priority was hernia risk.
3. Ranking of research priorities varied by gender, age, underlying disease that led to a stoma, length of time with a stoma and type of stoma.
4. It is anticipated that these results will contribute towards further research about setting the research agenda for the study of stoma‐related concerns that impact quality of life.

## 1 INTRODUCTION

There are a number of conditions that as part of treatment may necessitate the formation of a stoma including colorectal cancer, diverticular disease, incontinence, ulcerative colitis and Crohn's disease. Each year, for instance, 77% (n≈3000) of people in the United Kingdom undergoing anterior resection for rectal cancer will have a defunctioning or end stoma formed and 27% will still have a stoma at 18‐month follow‐up.^1^ A stoma is an artificial opening in the bowel that has been made to bring the bowel onto the surface of the abdomen in order to divert the flow of faeces or urine. The three types of eliminating stomas are colostomy, ileostomy and urostomy, which can be temporary or permanent. Recent systematic reviews of empirical evidence suggest that a stoma has a negative impact on quality of life of the person with the stoma and that of their spouses.^2^, ^3^, ^4^ Hence, identifying factors associated with poor quality of life and recognizing effective solutions are important for people with a stoma. Moreover, stoma‐related problems causing the most concern for people with a stoma should arguably take precedence and be research priorities.

In the United Kingdom, there is a recognized need to include patients, carers and clinicians in setting research priorities.^5^ Recently, the membership of the Association of Coloproctology of Great Britain and Ireland (ACPGBI) was involved in a Delphi study to reach a consensus on prioritizing clinical research questions in colorectal disease.^6^ The study produced a list of 25 research questions that can be considered to reflect the clinical matters of greatest importance by this expert panel. A similar research priority setting exercise was conducted by the American Society of Colon and Rectal Surgeons.^7^ The top‐scoring ACPGBI non‐cancer questions related to prevention and treatment of colorectal complications, including anastomotic leakage, parastomal hernia and the need for a defunctioning stoma.^7^ Top‐scoring cancer‐related research questions were; “Is there a price to cancer survival after treatment for colon, rectal and anal cancer? What is the impact of treatment on quality of life? What level of poor function is justified to avoid a permanent stoma?”^7^ Hence, surgeons clearly perceive stoma and quality of life for people with colorectal disease as national research priorities.

The ACPGBI acknowledge that there is a need to involve patient support groups in developing these research questions and establishing whether the questions resonate with patients as well as clinicians.^7^ To address this, we conducted a pilot survey of people with experience of having a stoma to identify research priorities about stoma‐related quality of life. The findings of this pilot survey could then be used to inform the conduct of large‐scale studies about research priorities for people with a stoma. In conjunction with the research priorities identified by the ACPGBI, this could contribute towards identification of the focus of future grant applications and act as a guide for future investigators and funders. Ultimately, the purpose is to improve the quality of life of people with a stoma through use of evidence‐based practice based on research priorities set by patients.

## 2 METHODS

A pilot cross‐sectional survey to determine research priorities about stoma‐related quality of life was conducted over 3 months in 2016. A pilot survey is a preliminary survey used to gather information prior to conducting a survey on a larger scale. The survey was closed after this 3‐month period. It was administered online using Bristol Survey On‐line (BOS), which is an online service that allows researchers to develop, deploy and analyse an online survey (https://www.onlinesurveys.ac.uk). An online questionnaire is a well‐established method used in health research^8^ and was chosen as a practical way of quickly reaching people to conduct the survey. A University of Stirling research and ethics committee approved the study (REF SREC 15/16 Paper No 54 Version 2).

### 2.1 Recruitment

The inclusion criterion was people 16 years of age and over who currently have or have had a stoma for treatment for any medical condition. Several relevant UK charities (Colostomy Association, Ileostomy Association, Urostomy Association and Bowel and Cancer Research) included a link on their website and on social media (eg. Facebook) to the study's online questionnaire. The Colostomy Association, Urostomy Association and Ileostomy Association represent people with a stoma, providing support, advice and practical information and Bowel and Cancer Research is a charity funding research. Only people who indicated consent by ticking a consent box could access the questionnaire, which is an approach to obtaining consent used in previous online health research projects.^9^ The questionnaire was in English, and the sampling approach and recruitment procedure were chosen for convenience and are commensurate with the purpose and scale of the pilot study.

### 2.2 Variables

Single items were used to gather data on respondents' gender, age when first stoma was formed, underlying disease that led to a stoma, type of stoma, if still had a stoma, and length of time with a stoma. This information was gathered to examine, for example, whether females and males had different research priorities and so on.

Nine stoma‐related quality of life topics derived from the literature were listed, and people who completed the survey were requested to rank them in order of research priority (range 1‐9). 1 represented the most important research priority and 9 the least important. The nine stoma‐related quality of life topics were selected as follows: first, potential topics were identified from the literature about stoma‐related quality of life;^2^, ^3^, ^4^ second, topics were discussed at a meeting by the research team (ie. listed authors) and representatives from the Colostomy Association, Ileostomy Association, Urostomy Association and Bowel and Cancer Research and a final list of topics was agreed; third, an individual with a stoma who acted as a patient advisor for the study and the research team and representatives from the Colostomy Association, Ileostomy Association, Urostomy Association and Bowel and Cancer Research completed the survey, which included the final list of nine topics. The wording for the questionnaire was revised in the light of feedback. An open‐ended question was included so that people completing the survey could indicate any other issues and concerns that were not listed, relating to their stoma, that they believed ought to be a research priority. A copy of the final questionnaire is available in a supplementary file.

### 2.3 Analysis

The following characteristics of the people who completed the survey were summarized using descriptive statistics: gender, age when first stoma was formed, underlying disease that led to a stoma, type of stoma, if still had a stoma, and length of time with a stoma. The proportion of people ranking each of the nine listed stoma‐related quality of life topics as most important (1 = most important) to least important (9 = least important) were calculated. Distributions of the priority scores for each of the nine research topics were examined and the median scores (range 1‐9) obtained. Research topics were then ranked according to the median scores, to indicate the order of priorities. Differences in the ranking of the nine research topics and the characteristics of people completing the survey (eg. gender, age when first stoma was formed, underlying disease that led to a stoma, type of stoma, if still had a stoma, and length of time with a stoma) were examined. The Mann–Whitney test was used to explore differences by age (2 groups: <=50 years old, <50 years old) or gender (2 groups: male, female). The Kruskal–Wallis test was used to explore differences by underlying disease (categorized into five groups: bladder cancer, colon cancer, rectal cancer, inflammatory bowel disease, other), type of stoma (3 groups: colostomy, ileostomy, urostomy) or length of time with stoma (4 groups: <=12 months, 13‐24 months, 25‐48 months and >48 months). The additional research priority topics identified by patients were listed and the proportion of people identifying each topic was calculated.

## 3 RESULTS

### 3.1 Characteristics of people completing the survey

There were 225 people completing the survey (Table 1). One hundred and sixty‐four were female (73%). Sixty‐nine (30.6%) were 61 years of age and over when they first had a stoma. Two hundred and fourteen (95.5%) still had a stoma with 90 (40.7%) of people having a stoma for ≥4 years. Ninety‐nine (44.4%) had a colostomy, 84 (37.7%) ileostomy and 40 (17.9%) a urostomy.

**Table 1:** Characteristics of people completing the survey

Characteristics	n=225 (%)
Gender	n=223
Male	59 (26.4)
Female	164 (73.6)
Age when first had stoma	n=225
<15 y	8 (3.6)
16‐20	4 (1.8)
21‐30	30 (13.3)
31‐40	29 (12.9)
41‐50	32 (14.2)
51‐60	53 (23.6)
61‐70	48 (21.3)
71‐80	18 (8)
Over 81	3 (1.3)
Reason for first stoma	n=222
Inflammatory Bowel Disease (eg. diverticular disease, Crohn's disease, ulcerative colitis)	80 (36)
Colon cancer	22 (9.9)
Rectal cancer	26 (11.7)
Bladder cancer	27 (12.2)
Birth defect	6 (2.7)
Peritonitis	3 (1.4)
Childbirth complications	2 (0.9)
Do not know	2 (0.9)
Other	54 (24.3)
Stoma at time of survey?	n=224
Yes	214 (95.5)
No	10 (4.5)
Type of stoma	n=223
Colostomy	99 (44.4)
Ileostomy	84 (37.7)
Urostomy	40 (17.9)
Length of time with stoma	n=221
0‐6 mo	16 (7.2)
7‐12	27 (12.2)
13‐18	23 (10.4)
19‐24	19 (8.6)
25‐36	27 (12.2)
37‐48	19 (8.7)
More than 4 y	90 (40.7)

### 3.2 Research priorities

Table 2 presents the proportion of respondents ranking each research topic. The table shows for instance, that “pouch leak problems and stoma bag/appliance problems” was ranked the most important research priority by 49.5% (n=104) respondents and hernia risk was ranked as the most important research priority by 21.3% (n=44) of people completing the survey.

**Table 2:** Proportion of respondents ranking each research priority

	Ranking n (%)								
Topic	1st	2nd	3rd	4th	5th	6th	7th	8th	9th
Pouch leak/appliance problems	104 (49.5)	30 (14.3)	19 (9)	10 (4.8)	16 (7.6)	5 (2.4)	9 (4.3)	7 (3.3)	10 (4.8)
Hernia risk	44 (21.3)	53 (25.6)	22 (10.6)	13 (6.3)	16 (7.7)	8 (3.9)	18 (8.7)	20 (9.7)	13 (6.3)
Physical activity	2 (1)	10 (5.1)	24 (12.2)	34 (17.3)	30 (15.2)	34 (17.3)	23 (11.7)	21 (10.7)	19 (9.6)
Pain and feeling uncomfortable	9 (4.5)	23 (11.6)	31 (15.6)	31 (15.6)	33 (16.6)	27 (13.6)	23 (11.6)	14 (7)	8 (4)
Smell and odour problems	12 (6)	24 (11.9)	27 (13.4)	31 (15.4)	29 (14.4)	24 (11.9)	21 (10.4)	14 (7)	19 (9.5)
Body image and body confidence	12 (6.2)	16 (8.2)	22 (11.3)	28 (14.4)	29 (14.9)	35 (17.9)	22 (11.3)	21 (10.8)	10 (5.1)
Fatigue and low energy levels	12 (6.3)	14 (7.4)	23 (12.2)	25 (13.2)	17 (9)	23 (12.2)	31 (16.4)	24 (12.7)	20 (10.6)
Sex life and intimacy	3 (1.6)	18 (9.4)	18 (9.4)	14 (7.3)	7 (3.6)	23 (12)	23 (12)	41 (21.4)	45 (23.4)
Health professional communication about living with a stoma	14 (7)	14 (7)	15 (7.5)	15 (7.5)	23 (11.6)	15 (7.5)	21 (10.6)	32 (16.1)	50 (25.1)

Using the median as an indicator of research priority it is possible to rank the nine topics in order of five research priorities. The most important research priority was pouch leak problems and stoma bag/appliance problems (median = 2). The second research priority was hernia risk (median = 3). Four topics were ranked as the third most important research priority: physical activity, pain and feeling uncomfortable, smell and odour problems, body image and body confidence (median = 5). The fourth research priority was fatigue and low energy levels (median = 6). Two subjects were ranked as the fifth most important research priority: sex life and intimacy and health professional communication about living with a stoma (median = 7).

### 3.3 Differences between groups in ranking research priorities

#### 3.3.1 Gender

There were significant differences in ranking research priorities between males and females for one research topic. Males ranked hernia risk as a more important research priority than women (mean rank 86.10 vs 109.84; Mann–Whitney *U‐test* = 3195, *P*<.05). No other differences were observed.

#### 3.3.2 Age

There were significant differences in ranking research priorities by age for two research priority topics. Those who were older when they first had a stoma (>50 years) ranked hernia risk as a more important research priority than those who were younger when they first had a stoma (<=50 years) (mean rank 91.16 vs 118.85; Mann–Whitney *U‐test* = 3902.5, *P*< .05). Those who were younger (<50 years) ranked physical activity as a more important research priority than those who were older when they first had a stoma (>50 years) (mean rank 88.84 vs 108.08; Mann–Whitney *U‐test* = 3891.5, *P*< .05).

#### 3.3.3 Underlying disease that led to a stoma

A Kruskal–Wallis test (χ^2^(4) = 13.2, *P*<.05) showed significant differences between the groups. People with Inflammatory bowel disease ranked fatigue and low energy levels as a more important research priority than any other group, with a mean rank of 77.89, compared with 95.08 for bladder cancer, 91.33 for colon cancer, 110.75 for rectal cancer and 109.42 for other reasons.

#### 3.3.4 Stoma type

Kruskal–Wallis tests showed that there were significant differences in ranking research priorities by stoma type for five research priority topics. People with a colostomy (mean rank 92.77) and urostomy (mean rank 90.91) ranked hernia risk as a more important research priority than those with an ileostomy (mean rank 123.01) ((χ2_(2)_ = 13.30, *P*<.05). People with an ileostomy (mean rank 88.44) and urostomy (mean rank 90.90) ranked physical activity as a more important research priority than those with a colostomy (mean rank 110.88) (χ2_(2)_ = 7.23, *P*<.05). People with a colostomy (mean rank 82.60) ranked smell and odour problems as a more important research priority than those with an ileostomy (mean rank 113.42) and urostomy (mean rank 122.76) (χ2_(2)_ = 17.49, *P*<.05) People with a urostomy (mean rank 82.31) ranked body image and body confidence as a more important research priority than those with an ileostomy (mean rank 91.59) and colostomy (mean rank 109.59) (χ2_(2)_ = 7.38, *P*<.05). Those with an ileostomy (mean rank 80.27) ranked fatigue and low energy levels as a more important research priority than those with a colostomy (mean rank 104.98) and urostomy (mean rank 102.4) (χ2_(2)_ = 8.08, *P*<.05).

#### 3.3.5 Length of time with a stoma

Kruskal–Wallis analysis showed that there was a significant difference in ranking research priorities by length of time with a stoma for one research topic. People who had a stoma for <=12 months (mean rank 90.68) or for >48 months (mean rank 88.08) ranked pain and feeling uncomfortable as a more important research priority than others (mean rank 120.71 for 13‐24 months and 106.60 for 25‐48 months).

### 3.4 Additional topics

Fifty‐four percentage (n=121) of people who completed the survey identified additional topics as research priorities. A minority (n=4) listed more than one additional topic. Topics that were identified by ≥5 people completing the survey are presented in Table 3. It is clear that most people who identified further topics focus on the physical problems caused by a stoma such as skin problems and flatulence. Topics that were identified by ≤5 people completing the survey are as follows: public awareness and perceptions of stoma, information about reversal, impact on family, hernia, exercising, returning to work, early diagnosis and cure, clothing and support garments, pregnancy and weight loss.

**Table 3:** Additional topics

Topic	N= (%)
Flatulence, constipation, pancaking, blockages, mucus, and anal discharge	25 (11)
Skin‐related problems (eg. soreness, itching and infection and fistulas)	22 (10)
Stoma bags and self‐management (eg. product design, adhesives and maintaining privacy)	20 (9)
WC facilities	10 (4)
Support at home and long‐term aftercare	7 (3)
Mental health and emotions	5 (2)
Diet and nutrition	5 (2)
Communication with health professionals	5 (2)

## 4 DISCUSSION

This survey produced a ranking of priorities for stoma‐related quality of life research of greatest important to people with a stoma. This is the first attempt to specifically engage people with a stoma to decide on research priorities using an online survey. The ACPGBI recommended the involvement of patients in developing research priorities.^7^ This study did this through a United Kingdom pilot survey of people with a stoma. The research priorities identified in this pilot study represent the perspectives of people with a stoma; 95% of those who replied still had a stoma.

The ACPGBI wished to find out if the research questions identified by clinicians would be supported by patients.^7^ Our survey suggests that they would be. The ACPGBI highlighted anastomotic leakage, parastomal hernia and quality of life as key research priorities. Our survey identified pouch leak problems and stoma bag/appliance problems as the top stoma‐related research priority and hernia risk as the second most important research priority. Prioritization should be given to these topics.

The main stated research priority identified in this study was pouch leak and stoma/bag appliances. Pouch leakage can cause discomfort and distress and the fear of this happening can profoundly affect daily life, activities, and social life.^10^ Modern stoma appliances have improved flatus filters and there are a variety of stoma accessories now available on prescription to secure the appliance and reduce risk of leaks. Nevertheless, the Ostomy Life Study (4000 participants from 11 countries) indicates that the majority of people with a stoma have issues related to leakage and ballooning.^11^ There is also strong evidence that peristomal skin complications, which cause leakage, are endemic.^12^

The second top research priority identified in this pilot survey was hernia risk. The reported incidence of parastomal herniation is variable, ranging from 10%‐52%.^13^ The reasons why a colostomy appears twice as likely to herniate as an ileostomy are not fully known beyond possibly the increased trephine diameter.^14^ What is known is that parastomal herniation not only causes further change in body image and cosmesis and increased pain but also a difficulty with stoma appliance application resulting in increased risk of pouch leakage which can result in impaired quality of life.^15^ Two large trials about surgical procedures to reduce the risk of parastomal herniation are underway in the United Kingdom.^16^, ^17^ There have only been a handful of education and abdominal exercise programmes in the post‐surgical period designed to prevent and support those with parastomal hernia.^13^, ^18^

Our pilot survey found differences in ranking of research priorities by gender, age, underlying disease that led to a stoma, length of time with a stoma and type of stoma. These differences may reflect variation in problems and concerns experienced by different groups of people with a stoma. One study for instance, found that female colorectal survivors with stomas had more specific psychological and social issues than men including diet management, physical activity, sexuality and social support.^19^ A comparison of quality of life in cancer and non‐cancer patients with colostomies found that non‐cancer patients reported lower scores for fatigue, strength, aches and pains, appearance and body image and skin irritation compared to cancer patients with colostomies.^20^ However, the study found that concerns associated with negative quality of life are common to *all* colostomy patients and include sexual problems, gas, constipation, travel difficulties and dissatisfaction with appearance. All of the research priorities presented in this article are therefore likely to resonate with many people with a stoma even if their ordering of research priorities varies.

The research priorities that we found in this pilot study may be an indicator of lack of support to address these concerns. Forty‐one percentage of survey respondents had been living with a stoma for at least 4 years and yet still identified pouch leak and stoma/bag appliances as the most pressing research priority. Research has identified complications after ostomy surgery as high as 21%‐70% including late complications such as peristomal dermatitis, hernia and stenosis.^21^ Hence, addressing the research priorities reported in this study is crucial.

Several people completing the survey identified additional topics as research priorities. Arguably, some of these topics could be categorized under the pre‐set list of topics; for example, skin problems could fall within the category, “pouch leak problems and bag/appliance problems.” However, the additional topics listed by people with a stoma highlight the topics or choice of wording that are perhaps more relevant and should therefore be considered for use in future research about research priorities.

### 4.1 Limitations

There is a risk that our list of nine stoma‐related quality of life topics missed key topics for people with a stoma and only reflect those issues deemed important by the research team and the collaborators. However, the inclusion of an open question whereby people completing the survey could identify additional topics partially addressed this limitation and the most common additional topics could be included in future surveys. It must be acknowledged that this is not a large sample and there will be a selection bias in our pilot study based on the surveyed population drawn from charities, who may have accessed charitable support to address any unmet emotional and practical support needs. In this anonymous web‐based pilot survey, we did not have access to medical record information, which limits our ability to describe the clinical characteristics of those surveyed. Certain people with a stoma, for example, males are under‐represented in the pilot survey. The organizations advertising the survey were United Kingdom charities. Nevertheless, we did not ask people completing the survey to indicate where they lived, which means we cannot be certain that most were from the United Kingdom. Finally, because it is a survey advertised using social media, we do not know the total population size and are therefore not able to examine whether the study is representative. To address these limitations, any future large‐scale survey could recruit from bowel cancer consultant patient lists so that we can calculate, for example the completion rate and study representativeness. Given these limitations, it is premature to claim that certain groups of people with a stoma will have different research prioritizations. Further research is therefore needed to replicate the findings in a more diverse population of people with a stoma.

## 5 CONCLUSIONS

This pilot survey suggests that people with a stoma are willing to engage in and set research priorities about stoma‐related quality of life. It is anticipated that these results will contribute towards further research about setting the research agenda for the study of stoma‐related concerns that impact quality of life. The question remains as to how this information should be disseminated and used by research funding bodies and researchers.
